# Continuing burden of HIV late presenters in the North East of England 2009

**DOI:** 10.1186/1758-2652-13-S4-P107

**Published:** 2010-11-08

**Authors:** N Premchand, K Golds, PY Tan, I Bittiner, N Sanker, ELC Ong

**Affiliations:** 1 Royal Victoria Infirmary, Infection & Tropical Medicine, Newcastle upon Tyne, UK; 2 University of Newcastle, Medical School, Newcastle upon tyne, UK; 3 Newcroft Centre, GUM, Newcastle upon Tyne, UK

## Purpose of the study

To determine whether the pattern of late presentation noted previously in 2007 and 2008 in all patients newly diagnosed with HIV in our regional unit [1] has decreased since the publication of The UK National HIV Testing Guidelines in 2008 [2].

## Methods

A retrospective case-note audit was undertaken in the ID/GUM clinics for all patients who were newly diagnosed with HIV in 2009. Patients were characterised as late presenters if they presented with a CD4 count of less than 200 or an AIDS defining illness. Medical records were reviewed to determine whether the patients had previously been diagnosed with a clinical indicator disease as defined by the UK National HIV Testing Guidelines, 2008, which might have facilitated earlier diagnosis of HIV. These 2009 data were compared with previous 2007 and 2008 data.

## Results

See table 1

**Table 1:** 

Source	ID 2009	GUM 2009	ID 2007	GUM 2007	ID 2008	GUM 2008
No of Patients	32	11	35	26	46	14

Late presenters	17 (53%)	3 (27%)	63%	59%	31%	21%

Male Gender	23 (72%)	11 (100%)	57%	54%	81%	89%

MSM	12 (38%)	10 (91%)	17%	54%	35%	79%

White British	19 (59%)	9 (82%)	37%	65%	54%	79%

Black African	11 (34%)	2 (18%)	49%	15%	43%	14%

Initial CD4 < 200 cells/µl	16*(52%)	3 (27%)	NK	NK	NK	NK

Symptomatic Seroconversion	3 (9%)	1 ((9%)	NK	NK	NK	NK

Previous Indicator Diseases	16 (50%)	1 (9%)	50%	50%	35%	29%

AIDS at/prior to diagnosis	8 (25%)	0(0%)	31%	0%	28%	0%

Commenced HAART	21 (66%)	6 (55%)	NK	NK	NK	NK

## Conclusions

Significant numbers of patients (53% in ID; 27% in GUM) still present with advanced HIV disease in the North East of England in 2009 despite the publications of the National UK Testing Guidelines in 2008. The numbers of late presenters have not changed as compared to 2007 and 2008. This is despite that a large proportion having had previous indicator diseases that should have prompt clinicians to test for HIV. Further education and awareness of the UK National Testing Guidelines 2008 should be encouraged if this burden of late presenters is to be reduced.
